# Diabetic Charcot Neuroarthropathy: A Contemporary Review of Molecular and Genetic Markers

**DOI:** 10.1177/10711007251390516

**Published:** 2025-12-16

**Authors:** Gyan Narayan, Vinod K. Panchbhavi

**Affiliations:** 1 John Sealy School of Medicine, The University of Texas Medical Branch, Galveston, TX, USA; 2 Department of Orthopaedic Surgery and Rehabilitation, The University of Texas Medical Branch, Galveston, TX, USA

**Keywords:** biomarkers, Charcot neuroarthropathy, diabetes mellitus, foot diseases, genetic markers

## Abstract

**Graphical Abstract:**
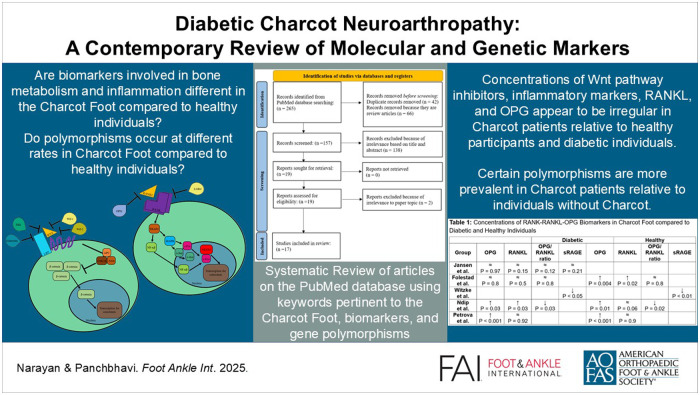
This is a visual representation of the abstract.

This is a visual representation of the abstract.

## Introduction

Charcot neuroarthropathy, also known as Charcot foot (CF), is a complication of diabetes mellitus and other disorders characterized by peripheral neuropathy. Its prevalence ranges from 0.1% to 29% among patients with diabetes and appears to be increasing.^52^ The prevalence rises to 35% in patients with peripheral neuropathy.^13^ Jean-Martin Charcot discovered this condition in the 19th century when he described arthropathy in the lower extremity with myelopathy.^1^ CF is characterized by inflammation, osteolysis, and subsequent fractures and dislocation.^44^ CF is characterized by redness, edema, and a temperature greater than 2 °C higher than the contralateral foot.^28^ The tarsometatarsal and tarsal joints are most frequently affected.^46^ Many patients with CF experience pain of relatively low intensity. The low specificity of symptoms in CF (pain, erythema, heat) often leads to incorrect diagnoses, such as osteomyelitis.^4^ Several major theories attempt to describe the pathogenesis of CF.^9^ The neuro-bone-inflammatory theory implicates inflammatory factors in the progression of CF. In the neurotraumatic theory, repeated injury to Charcot-affected joints leads to complex fractures.

The Eichenholtz classification system is most often used to determine the progression of CF and can assist in the selection of appropriate treatment.^45^ The most established treatment for CF is offloading of the affected foot using a total contact cast (TCC).^48^ Progression of CF can result in bone deformities, including the rocker-bottom deformity.^32^ A stable deformity can be managed without reconstruction with an ostectomy or exostectomy.^7^ Reconstruction with correction of deformity can be done with a variety of measures, including internal and external fixation.^22^ Amputation may be needed when reconstructive and nonreconstructive options are no longer viable.^54^

**Figure 1.**
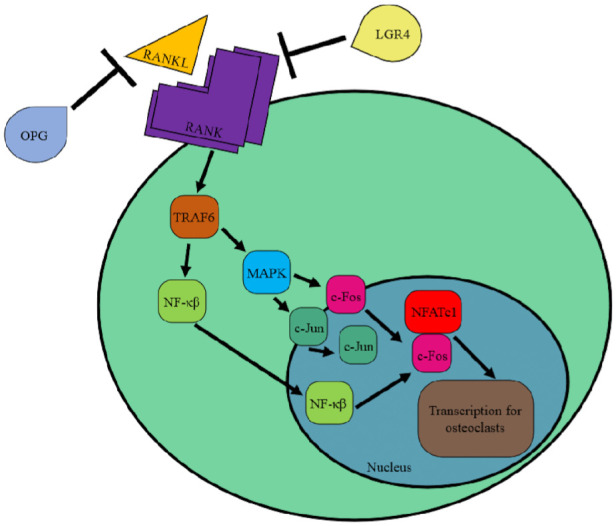
Overview of RANKL-RANK signaling pathway.

The RANKL-RANK-OPG signaling pathway for osteoclastogenesis^10^ is believed to be heavily involved in the development of CF.^31^ The receptor activator of nuclear factor-κβ ligand (RANKL) binds and activates the receptor activator of nuclear factor-κβ (RANK), causing it to cluster and recruit TNF receptor–associated factor 6 (TRAF6) (Figure 1). TRAF6 then activates NF-κβ and mitogen-activated protein kinase, which translocate c-Fos and c-Jun to the nucleus.^23^ NF-κβ provides a costimulatory signal for C-Fos, leading to transcription for osteoclastogenesis with nuclear factor of cytoplasmic activated T cells (NFATc1). Osteoprotegerin (OPG) and LGR4 are inhibitors of RANK signaling.^30,50^ Increases in RANKL have been found in the bone marrow of women with osteoporosis.^49^ Imbalance in this pathway may contribute to arthritis, osteolysis, and prosthesis loosening.^51^

**Figure 2.**
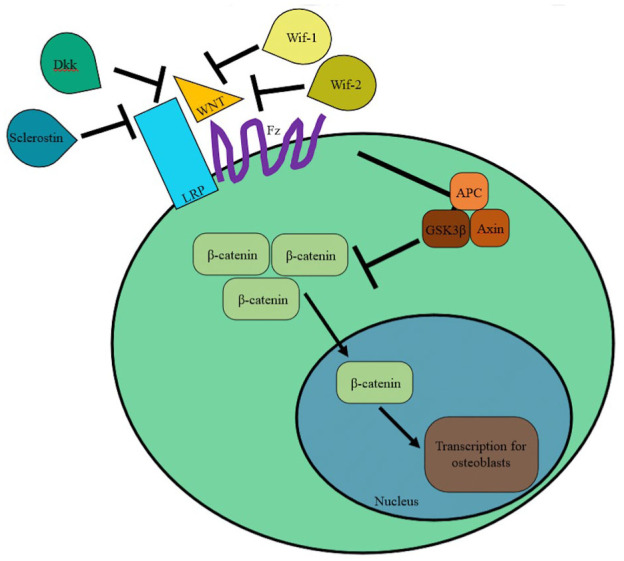
Overview of Wnt/β-catenin signaling pathway.

The Wnt pathway is thought to have some impact on Charcot pathogenesis.^1^ Wnt binding to the cell membrane receptor Frizzled (Fz) leads to complexation with low-density lipoprotein receptor-related proteins 4, 5, and 6 (LRPs), which prevents the degradation of β-catenin in the cytosol by Axin, GSK3β, and adenomatous polyposis coli (APC) (Figure 2). β-Catenin subsequently accumulates and translocates to the nucleus, leading to proliferation and differentiation of osteoblasts and osteoclast suppression.^19^ Sclerostin, Dickkopf (Dkk), Wnt-inhibitory factor-1, and factor-2 (Wif-1 and Wif-2) inhibit Wnt/β-catenin signaling. Decreases in sclerostin and Dkk were found to promote bone fracture healing.^1^ Increased sclerostin is found in osteoarthritis, and overactivation of Wnt leads to osteophyte formation in spondylarthritis.^55^

The aim of this literature review is to summarize recent findings on biological and genetic markers in CF.

## Methods

**Figure 3.**
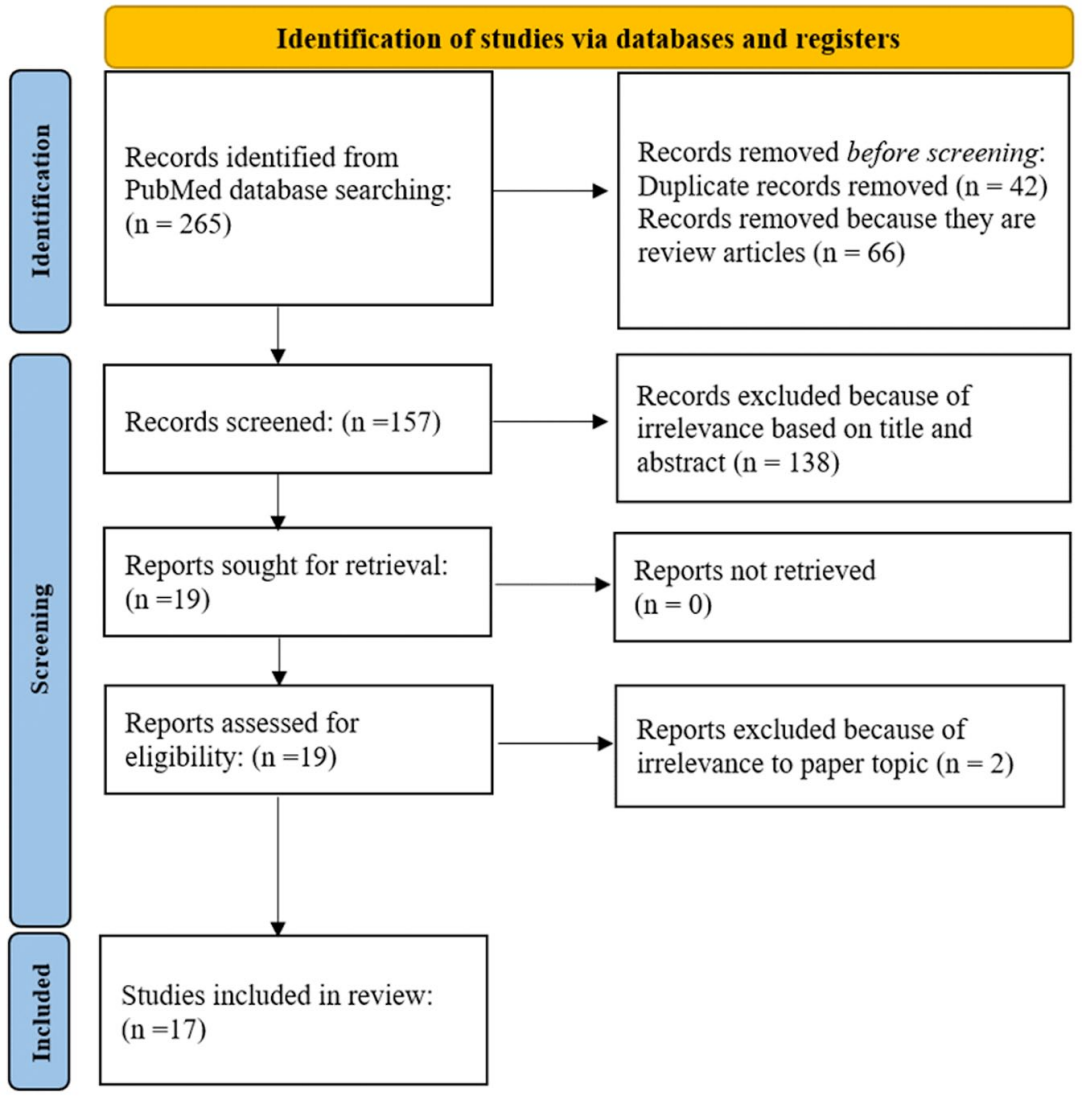
PRISMA diagram of study screening process.

The Preferred Reporting Items for Systematic reviews and Meta-Analyses (PRISMA) guidelines were used to identify eligible articles for the literature review (Figure 3). One author (G.N.) searched PubMed across all years using the criteria “biomarkers AND Charcot foot,” “cytokines AND Charcot foot,” “gene AND Charcot foot” for full-text articles using clinical trials, randomized control trials, and experimental papers. The inclusion criteria were original articles. Nonoriginal articles and review articles were excluded. Relevant studies were assessed based on their titles and abstracts. After screening was completed, a total of 17 articles remained and were analyzed.

## Results

### Markers in the RANKL-RANK-OPG Signaling Pathway

Patients with acute CF have increased osteoclast formation and bone resorption compared with diabetic participants (DP) and healthy participants (HP), as a result of the RANKL pathway.^31^ Osteoclasts generated in CF were found to have greater activity after the addition of sRANKL, compared with the DP and HP. Furthermore, the addition of excess OPG decreased bone resorption in CF, DP, and HP.

OPG concentrations do not differ between CF and DP (Table 1).^26^ This is in consensus with previous research, which found that OPG levels are higher in CF and DP compared with HP; there is no significant difference between the CF and DP in the acute phase.^17^ Additionally, OPG levels are increased in cases of diabetic neuropathy, possibly because of unnoticed bone injury and subsequent increased remodeling activity.^6,36^ The increased OPG levels in CF and DP imply amplified osteoblast activity in these groups compared with HP. After 2 years of TCC treatment in CF, OPG levels were higher in DP than in CF and HP, suggesting a decrease in bone remodeling activity in CF.^17^

**Table 1.** Relative Concentrations of RANKL/RANK-Associated Biomarkers in Acute Charcot Foot Compared to Diabetic and Healthy Individuals.

	Diabetic				Healthy			
Group	OPG	RANKL	OPG/RANKL ratio	sRAGE	OPG	RANKL	OPG/RANKL ratio	sRAGE
Jansen et al^27^	≈ *P* *=* .965	≈ *P* *=* .148	≈ *P* *=* .156	≈ *P* *=* .212				
Folestad et al^17^	≈ *P* *=* .8	≈ *P* *=* .5	≈ *P* *=* .8		↑ *P* *=* .004	↑ *P* *=* .02	≈ *P* *=* .8	
Witzke et al^53^				↓ *P* < .05				↓ *P* < .01
Ndip et al^35^	↑ *P* *=* .031	↑ *P* *=* .033	↓ *P* *=* .033		↑ *P* *=* .01	≈ *P* *=* .055	↓ *P* *=* .019	
Petrova et al^41^	↑ *P* < .001	≈ *P* *=* .915			↑ *P* < .001	≈ *P* *=* .915		

Abbreviations: OPG, osteoprotegerin; RANKL, receptor activator of nuclear factor-κβ ligand; sRAGE, soluble receptor for advanced glycation end products.

Furthermore, plasma RANKL levels are significantly higher in CF and DP relative to HP and are similar between DP and CF at the initial measurement.^17^ However, after 2 years of TCC, plasma RANKL levels are significantly higher in DP compared with both CF and HP. Despite the differences in concentration of each of these proteins, the ratio of OPG/RANKL is similar between the 3 groups both during the initial measurement and after 2 years. These findings contradict later research, which found no difference in the soluble RANKL level between patients with CF and diabetic neuropathy in the acute phase but that soluble RANKL concentration decreases in CF during an 8-year period, leading to an increase in the OPG/RANKL ratio over this time.^26^ The discrepancy in measured OPG/RANKL ratio could be due to the difference in time periods (2 years vs 8 years). In an earlier study with a smaller sample size, OPG concentrations were increased in CF compared with both DP and HP, and the ratio of OPG/RANKL was decreased in CF compared with DP and HP in the acute phase.^35^ Additionally, there are no significant differences in in vivo gene expression of RANKL or OPG.^8^ These contradicting findings could result from differences in acuteness of CF presentation.

The circulating soluble receptor for advanced glycation end products (sRAGE) concentration is decreased^53^ or similar^26^ in CF relative to DP and HP. Advanced glycation end products (AGEs) are products of hyperglycemia and oxidative stress, and sRAGE is induced by inflammation. AGEs activate the RANKL pathway by binding to RAGE, inducing osteoclastogenesis.^18^

### Markers in the Wnt Signaling Pathway

Sclerostin levels are significantly higher in DP compared to CF and HP initially and after 2 years (Table 2).^17^ Dkk-1 levels are initially higher in DP compared with CF and HP. After 2 years, the Dkk-1 levels in DP are higher than in HP but not CF with TCC. But Dkk-1 levels are not significantly different between CF and HP at this time. Wnt-1 levels are higher in DP relative to healthy subjects and CF both initially and after 2 years. Wif-1 levels are similar between CF and DP, and both groups had Wif-1 levels significantly higher than HP at inclusion. After 2 years of TCC treatment, the Wif-1 levels are still higher in both CF and DP relative to HP and similar between CF and DP. Wif-1 negatively regulates osteoblast differentiation and maturation in the Wnt pathway. These findings suggest decreased inhibition of osteoblastogenesis in CF compared with DP, which may serve to compensate for increased osteoclast activity.

**Table 2.** Relative Concentrations of Wnt/β-Catenin-Associated Biomarkers in Acute Charcot Foot Compared to Diabetic and Healthy Individuals.

	Diabetic				Healthy			
Group	Sclerostin	Dkk-1	Wnt-1	Wif-1	Sclerostin	Dkk-1	Wnt-1	Wif-1
Folestad et al^17^	↓ *P* *=* .01	↓ *P* *=* .003	↓ *P* *=* .03	≈ *P* *=* .09	≈ *P* *=* .4	≈ *P* *=* .7	≈ *P* *=* .7	↑ *P* *=* .004

Abbreviations: Dkk, Dickkopf; Wif-1, Wnt-inhibitory factor-1; Wif-2, Wnt-inhibitory factor-2.

### Inflammatory Markers

CF is associated with differing levels of various inflammatory markers in the initial phase compared with HP and DP (Tables 3 and 4). Subjects with CF have lower plasma levels of the proinflammatory cytokine IL-17F than HP and DP with neuropathy.^15^ At a 2-year follow-up after treatment with TCC, the levels of IL-17F are not significantly different between CF and DP or HP. During the acute phase, levels of plasma IL-17E do not differ between CF, DP with neuropathy, or HP. However, at a 2-year follow-up, IL-17E levels are significantly higher in CF compared with the other subjects. The levels of plasma IL-17A do not significantly differ between CF, DP, or HP at inclusion or after 2 years.

**Table 3.** Relative Concentrations of Inflammatory Markers in Acute Charcot Foot Compared to Diabetic Individuals.

	Folestad et al^16^	Folestad et al^17^	Petrova et al^41^	Pasquier et al^40^
IL-17F	↓ *P* *=* .024			
IL-17E	≈ *P* *=* .073			
IL-17A	≈ *P* *=* .77			
IL-6		≈ *P* *=* .64	↑ *P* *=* .002	
IL-8		≈ *P* *=* .83		
TNF-α		↓ *P* *=* .005	↑ *P* *=* .010	
IL-2				↑ *P* < .001
IL-16				≈ *P* > .05
IL-1RA		≈ *P* *=* .45		↑ *P* < .01
IL-1β		↓ *P* *=* .029	≈ *P* *=* .254	
IL-1RA/ IL-1β		↑ *P* *=* .007		
CRP			↑ *P* *=* .007	
C-terminal telopeptide			↑ *P* *=* .004	
Bone alkaline phosphatase			↑ *P* *=* .006	
Tartrate-resistant alkaline phosphatase			≈ *P* *=* .126	
G-CSF				↑ *P* < .001
GM-CSF				↑ *P* < .001

Abbreviations: CRP, C-reactive protein; G-CSF; granulocyte colony-stimulating factor; GM-CSF, granulocyte macrophage colony-stimulating factor; IL, interleukin; TNF-α, tumor necrosis factor alpha.

**Table 4.** Relative Concentrations of Inflammatory Markers in Acute Charcot Foot Compared With Healthy Individuals.

	Folestad et al^16^	Folestad et al^17^	Petrova et al^41^	Pasquier et al^40^
IL-17F	↓ *P* *=* .047			
IL-17E	≈ *P* *=* .073			
IL-17A	≈ *P* *=* .77			
IL-6		≈ *P* *=* .64	↑ *P* *=* .002	
IL-8		≈ *P* *=* .83		
TNF-α		≈ *P* *=* .64	↑ *P* *=* .010	
IL-2				↑ *P* < .001
IL-16				≈ *P* > .05
IL-1RA		↑ *P* *=* .004		↑ *P* < .01
IL-1β		≈ *P* *=* .29	≈ *P* *=* .254	
IL-1RA/ IL-1β		↑ *P* *=* .027		
CRP			↑ *P* *=* .007	
C-terminal telopeptide			↑ *P* *=* .004	
Bone alkaline phosphatase			↑ *P* *=* .006	
Tartrate-resistant alkaline phosphatase			≈ *P* *=* .126	
G-CSF				↑ *P* < .001
GM-CSF				↑ *P* < .001

Abbreviations: CRP, C-reactive protein; G-CSF; granulocyte colony-stimulating factor; GM-CSF, granulocyte macrophage colony-stimulating factor; IL, interleukin; TNF-α, tumor necrosis factor alpha.

CF is associated with an increased plasma concentration of interleukin (IL)-6, a proinflammatory cytokine, 4 months after initial measurement when wearing a TCC.^16^ However, the IL-6 concentration does not differ significantly from HP or DP initially or after 2 years. Additionally, arteriovenous flux of IL-6 is significantly higher in Charcot neuropathic feet compared with the non-Charcot neuropathic feet of the same patients approximately 2 months after symptom onset.^27^ This finding indicates that IL-6 is produced locally in the affected foot to increase inflammation. IL-6 concentration was recently found to have a greater predictive value than C-reactive protein (CRP) concentration for Charcot neuropathy.^11,41^

The pro-inflammatory cytokine tumor necrosis factor alpha (TNF-α) is higher in CF 4 months after inclusion but not 2 years after inclusion when wearing a TCC.^16^ This aligns with past findings of moderate or diffuse presence of IL-6 and TNF-α in tissue samples obtained from the affected foot of Charcot patients.^2^ IL-6 and TNF-α are both associated with increased osteoclastogenesis.^14^ Additionally, an in vitro study demonstrated that anti-TNF-α antibody decreases osteoclastic bone resorption in CF when treated with macrophage colony-stimulating factor and RANKL, suggesting that TNF-α plays a role in acute Charcot pathogenesis.^42^

Patients with CF have higher levels of CRP, IL-6, and TNF-α, as well as the bone turnover markers C-terminal telopeptide, bone alkaline phosphatase, and OPG in their forearm veins compared with HP and DP at initial measurement.^41^ This finding appears to contradict previous work, which showed that OPG levels are higher in both CF and DP relative to HP, without a significant difference between CF and DP.^17^ This discrepancy could possibly be explained by the higher sample size used by the Folestad group. After 3 months of TCC treatment, there is a decrease in TNF-α but not CRP, IL-1β, C-terminal telopeptide, bone alkaline phosphatase, tartrate-resistant acid phosphatase, OPG, or soluble RANKL.^41^ There was no further reduction in TNF-α and IL-6 or other biomarkers at the time of resolution. Additionally, the inflammatory cytokines granulocyte colony-stimulating factor (G-CSF), granulocyte macrophage colony-stimulating factor (GM-CSF), IL-1RA, and IL-2 are increased in CF compared with DP and HP during the acute phase, which underscores the increased inflammation during the acute state.^40^

Plasma IL-1β concentration is higher for DP compared with CF and HP. However, the level of IL-1β is not significantly different between CF and HP. IL-1β is a pro-inflammatory cytokine associated with cartilage destruction in osteoarthritis, whose effect is compounded by TNF.^24^ Plasma IL-1RA levels are elevated in CF and DP compared with HP at inclusion and after 2 years.^16^ However, IL-1RA levels are higher in CF at 4 months after inclusion compared to at inclusion. The ratio of IL-1RA/IL-1β is significantly higher in CF compared with DP and HP at inclusion and after 2 years. IL-1RA is an anti-inflammatory cytokine, which inhibits IL-1β and osteoclast activity.^12^ The relatively high and normal concentrations of IL-1RA and IL-1β in CF, respectively, could indicate modulation of the inflammation caused by proinflammatory cytokines.

### Genetic and Epigenetic Factors

Genetic factors are associated with CF.^20^ Patients with active CF had decreased *WNT3A* and *WNT5A* gene expression compared with HP and DP. WNT3A is a canonical ligand in the Wnt pathway, which promotes increased osteoblast activity. WNT5A is a ligand in the noncanonical Wnt pathway, which also promotes osteoblastogenesis. Thus, these findings emphasize the attenuation of osteoblastic activity in CF. TCF7L2 is a transcription factor that foments osteoblast activity, and polymorphisms in *TCF7L2* are associated with type 2 diabetes mellitus.^21^ *TCF7L2* gene expression is decreased in both CF and DP compared to HP. Osteocalcin gene expression is decreased in both CF and DP relative to HP. Type 1 collagen and fibronectin gene expression are lower in both CF and DP relative to HP. However, β-catenin gene expression is not significantly lower in CF compared with HP, possibly because of its regulation at the posttranslational level in addition to at the transcriptional level.

One group found differences in gene polymorphisms in the *OPG* gene.^29,43^ DP with neuropathy but without CF had different rates of 1217C/T, 950T/C, and 245T/G polymorphisms in *OPG* compared with HP. Later research with a larger sample size has not shown the 950T/C polymorphism to be associated with CF.^3^ Significant differences were found between CF and DP with and without neuropathy for 1181G/C and 950T/C polymorphisms.^29^ There is significant linkage between the G allele and CF for the 1181G/C polymorphism.^43^ Lastly, significant differences were found between CF and DP in the 1217C/T and 245T/G polymorphisms.^29^ Moreover, the polymorphism 6890A/C in the *OPG* gene was found to be significantly different between CF, DP with neuropathy, and HP.^3^ However, a meta-analysis did not find any significant associations between either the 1181C/G or 950T/C polymorphism in CF and found significant associations only between the homozygous 245A/C polymorphism and CF.^37^ Additionally, the variants rs1872426, 950T/C, and rs1485286 in *OPG* were correlated with CF.^33^ Although the 1181G/C polymorphism occurs in an exon, 245T/G, 950T/C, and 6890A/C polymorphisms all occur in introns of *OPG.*

Furthermore, 290C/T, 643C/T, and 693G/C polymorphisms in the RANKL gene are associated with both CF and diabetic neuropathy.^3^ These polymorphisms are all in noncoding regions of RANKL. In RANKL 643C/T, the CT genotype and minor allele T had greater frequences in CF and DP with neuropathy compared to HP.^47^ Furthermore, RANKL 693C/G had an increased frequency of the homozygous mutant GG and minor allele G in DP with neuropathy and CF compared with HP. According to this study, the 643C/T polymorphism is significantly associated with neuropathy alone, whereas the 693C/G polymorphism is associated with both neuropathy and CF. Despite this study being more recent, it has a smaller sample size than that of the Bruhn-Olszewska group.

Whole methylome analysis reveals several differentially methylated genes in the circulating monocytes of CF compared to DP with and without neuropathy.^39^ These genes are mostly involved in monocyte migration and differentiation into osteoclasts. Additionally, 25 miRNAs are differentially expressed between CF and DP with and without neuropathy, of which 8 are involved in inflammatory or bone-related processes.^38^

## Discussion

This literature review summarizes findings on various biomarkers involved in the RANK-RANKL-OPG pathway, Wnt pathway, and inflammation. Additionally, recent findings on genetic and epigenetic preponderances in CF are included.

There have been conflicting findings regarding the relative concentration of OPG and RANKL in CF compared with DP; thus, further research should be conducted to further establish this relationship.^17,26,35,41,53^ The discrepancy in AGE concentration could be due to different sample sizes.^26,53^ The increase of OPG and RANKL in CF relative to HP supports the idea that the RANK-RANKL-OPG pathway is involved in CF. It also suggests a complex interplay between the promotion and inhibition of osteoclastogenesis.

Concentrations of inhibitors of the Wnt pathway, except Wif-1, are decreased in CF relative to DP but are similar between CF and HP, although this finding has not been replicated.^17^ This suggests that osteoblastogenesis is relatively normal in CF but is decreased in DP.

The initial reduction in IL-17F in CF aligns with the slowed bone formation during the initial phase, whereas the increase in IL-17F concentration over 2 years could indicate increases in bone formation following treatment.^15^ IL-17E suppresses osteoclastogenesis in rheumatoid arthritis.^32^ The relative increase in IL-17E concentration in CF after treatment compared to the other groups could be due to the need for continued osteogenesis and bone remodeling even after treatment is initiated. Additionally, the difference in concentration patterns between IL-17A and IL-17F is unexpected because both cytokines are structurally similar and regulated similarly.^15,25^ However, IL-17F is associated with increased expression of the mature bone markers Col1, Col2, BSP, and osteocalcin.^34^ Nevertheless, the paradoxical decrease in IL-17F relative to IL-17A in CF warrants further investigation. The finding that the anti-inflammatory IL-1RA concentration is increased in CF relative to HP suggests that the initial pro-inflammatory state is moderated to prevent excess bone breakdown.^16,40^

The 1181G/C polymorphism of *OPG* occurs in an exon, and 245T/G, 950T/C, and 6890A/C polymorphisms occur in introns.^37^ Additionally, only the homozygous 245A/C polymorphism is significantly associated with CF. These results complicate the impact of many of these polymorphisms on the *OPG* gene. These conflicting findings warrant further investigation of *OPG* gene polymorphisms and their effect on Charcot pathogenesis. Polymorphisms in *RANKL* may also be associated with Charcot pathogenesis, but more research must be conducted to evaluate each of their specific effects.^47^ Additionally, epigenetic modifications have been correlated with CF, but these studies have not yet been replicated.^38,39^

One significant weakness of this literature review is that all included articles reported biomarkers in CF after the diagnosis of CF had already been made. Thus, the available evidence would not allow biomarkers present before diagnosis to predict a patient’s risk of CF. Furthermore, additional risk factors such as peripheral artery disease could be confounders in the included studies.^5^ Another weakness of this literature review is that many measurements of biomarker concentrations have not been replicated. Additionally, much of the research on genetic polymorphisms was undergone in the Western hemisphere, but the frequency of polymorphisms varies across different regions.

## Conclusion

In summation, recent research has revealed much about the complex interplay between pro-inflammatory, anti-inflammatory, osteoclastic, and osteoblastic markers in CF. Additionally, recent findings have suggested possible genetic and epigenetic risk factors for Charcot development. However, there are still many unanswered questions, such as the concentration of inflammatory markers over several years and the impact of the LGR4 ligand on CF. Extremely limited research exists regarding the role of the Wnt pathway in Charcot pathogenesis, and studies of genetic risk factors for CF are very limited and have yielded conflicting results. Future exploration of these questions would ensure an earlier and more accurate diagnosis of CF. Additionally, further research into the change in concentrations of biomarkers over time would provide valuable insight into the efficacy of Charcot treatment, progress, and prognosis.

## Supplemental Material

sj-pdf-1-fai-10.1177_10711007251390516 – Supplemental material for Diabetic Charcot Neuroarthropathy: A Contemporary Review of Molecular and Genetic Markers Supplemental material, sj-pdf-1-fai-10.1177_10711007251390516 for Diabetic Charcot Neuroarthropathy: A Contemporary Review of Molecular and Genetic Markers by Gyan Narayan and Vinod K. Panchbhavi in Foot & Ankle International
